# Preventing road deaths through safe system design

**DOI:** 10.2471/BLT.21.021121

**Published:** 2021-11-01

## Abstract

A consensus is building around the value of systemic approaches to collision prevention. In Latin America, cities are beginning to take heed. Gary Humphreys reports.

It is the young motorcyclist with the dreadlocks that Dilia Puerto remembers. “It happened in the afternoon rush hour,” she says. “He came racing down the street, and a car hit him. He was slammed into a post and died right there on the sidewalk.”

The young man was one of 600 people killed in collisions in Bogotá, Colombia in 2020 and one of an estimated 1.35 million people killed worldwide that year. According to the Pan American Health Organization’s (PAHO) 2019 *Status of road safety in the Region of the Americas *report, motorcyclists account for nearly a quarter of all road traffic deaths in the region.

A taxi driver with 10 years’ experience working the streets of Bogotá, Dilia has seen her share of collisions, but few that were so predictable. “The young man was going much too fast,” she says. “And his helmet wasn’t even attached – it was just perched on the back of his head.”

From Dilia’s point of view, a view that would be shared by many people, it was the young man’s behaviour that led to his death.

As policymakers and researchers, worldwide, look back on the United Nations-backed Decade of Action for Road Safety, which ended in May 2021, they are questioning that view and asking themselves if focusing on the individual is the best way to draw up road safety policy.

For many of those experts, the answer is a resounding “no”. “We have been preaching to road users and prescribing better manners for decades. It doesn’t help, as indicated by the number of deaths on the world’s roads remaining stuck at around 1.35 million, despite the Decade of Action,” says Victor Pavarino, National Traffic Safety Advisor at PAHO’s Brazil country office.

“Limiting our focus to personal responsibility is not particularly helpful, and prevention policy derived from it, such as using awareness-raising and educational campaigns, has been shown to be ineffective if it is not aligned with infrastructure and enforcement measures,” he adds.

What does help, in Pavarino’s view, is the adaptation or transformation of the roads themselves as part of broader systemic reforms. “The truth is people make mistakes,” he says. “But that is not a conclusion. It is a starting point for Safe System design.”

Pavarino is first to admit that this concept is not new. In fact, its origins can be traced back to work done in Sweden in the 1970s which came to fruition in the Safe System approach, implemented by that country as part of a serious accident and death prevention initiative called Vision Zero that was launched in 1997.

“People make mistakes. This is not a conclusion. It is a starting point for Safe System design.”

Victor Pavarino

“The Safe System approach builds on public health frameworks such as the Haddon matrix which serve to identify the human, vehicle and equipment and environmental risk factors that contribute to a crash and subsequent injuries,” explains Dr Nhan Tran, head of Safety and Mobility in the World Health Organization Department of Social Determinants of Health.

For Tran, it is important to recognize that while the infrastructure and behavioural elements appear to be separate, they are in fact intertwined. “The Safe System approach not only constitutes an acknowledgement that human errors are inevitable, it also recognizes the fact that behaviours are a function of the system,” he says.

Claudia Adriazola-Steil, Director of Health and Road Safety at the World Resources Institute (WRI), a global research and analysis, non-profit organization, agrees, citing the example of four-lane highways flanked by “interminable” city blocks. “We all know what happens,” she says: “Traffic accelerates and if you have blocks that are 500 or 800 metres long with pedestrian crossings at the intersections, people will cross mid-block.”

WRI is working with public and private sector partners in multiple cities worldwide to change system design, and Adriazola-Steil sees increasing interest in Safe System approaches. “The conversations we are having now are different to the ones we had 10 years ago, and governments are starting to pay attention, including governments in low- and middle-income countries such as Brazil, Colombia, Ethiopia, Mexico, and India,” she says.

For the time being, however, it is city authorities, and city mayors, who are taking the lead. In Bogotá, for example, which is one of the first cities to implement Safe Systems policies in Latin American, local government is implementing a speed management programme which was launched in 2018.

According to Jessica Kisner, Road Safety Manager at WRI Colombia, WRI worked alongside PAHO country representatives and other partners to provide guidance and technical support for the programme, which was funded by the Bloomberg Initiative for Global Road Safety (BIGRS) and local government.

“There used to be around 2000 fatalities a year on the streets of Bogotá,” says Kisner. “That number came down in the wake of various reforms, but over the past 10 years has stagnated at around 500 to 600 road deaths per year. The speed management programme was part of an initiative designed to reduce traffic fatalities by 35% in a decade.”

The programme began by targeting five high-risk corridors identified using geo-referenced collision data and modelling. To date, 10 arterial corridors have been transformed and a city-wide 50-kilometre-per-hour (kph) speed limit imposed, except in three 60-kph corridors. “According to the transport ministry, 39 people were killed on Bogotá’s roads in 2019, representing a 26% decline compared to average fatalities reported between 2015 and 2018,” Kisner explains.

Getting local communities to embrace the changes was challenging. “Residents argued that the speed limits would make their commute longer and reduce the city’s productivity,” Kisner says. Using their surveillance and modelling capacity, WRI were able to show that the projected speed reduction on the arterial road with the most fatal accidents would only increase journey times by a maximum of 14 seconds. “Being able to cite solid data really helped,” Kisner adds.

Kisner also credits “tactical urbanism” with changing people’s attitudes – an approach characterized by localized, often community-led interventions that transform targeted areas. One such project is called Plazoletas, which consists of 11 locations where just over 16 000 square metres of street were turned into pedestrian-friendly streets with traffic calming infrastructure such as speed bumps, but also cycle lanes, wide sidewalks, benches and planters.

According to Pavarino similar initiatives have been launched in the cities of Salvador and Campo Grande. The Campo Grande project was implemented in 2020 and involved the introduction of new paving, street furniture and landscaping. Overhead power lines were removed and re-installed underground.

“Behaviours are a function of systems.”

Nhan Tran

But can small-scale localized initiatives really make a difference? Pavarino believes they can, arguing that they not only allow for the testing and improvement of interventions – they also give policy-makers a chance to gauge public support. Kisner agrees, pointing out that Bogotá’s speed management programme was executed in phases in order to better understand the speed management dynamics and impacts, while also gauging community support. For Kisner the fact that the programme, initiated by Bogotá’s former mayor, Enrique Peñalosa, was continued and scaled up by the incumbent, Mayor Claudia Lopez, is indicative of widespread support and political engagement.

However, both Kisner and Pavarino acknowledge the challenges faced in persuading national government to get behind Safe System initiatives. Those challenges start with convincing the different agencies that have a stake in national road systems to rethink their priorities.

Natalia Tinjacá Mora, a national road safety expert at PAHO who previously worked in Colombia’s National Road Safety Agency and is coordinating BIGRS projects in the country, offers some valuable insights: “To advance the Safe System agenda, you need intersectoral dialogue,” she says. “From the finance ministry that is footing the bill, and the designers who are planning the roads to the police who have a vital responsibility in traffic control and regulatory enforcement, and the public health authorities that are trying to bring down the serious injury and mortality rates. They all have to come together, and that can be challenging.”

According to Tinjacá, impediments to dialogue include major differences in priorities. “Road planners still put flow, speed and efficiency, before road safety,” she says. “They see speed not just as proof of the system’s efficient operation but as a development issue. I have had high-level people say to me, ‘Why are you trying to slow the country down? We need to go faster’.”

For Pavarino, such obstacles have tended to result in a default response: blame the individual. “At the heart of the Safe System approach is the notion that responsibility for road safety should be shared by all parties, including road users and system designers. By blaming the individual, road traffic authorities abdicate responsibility,” he says.

**Figure:**
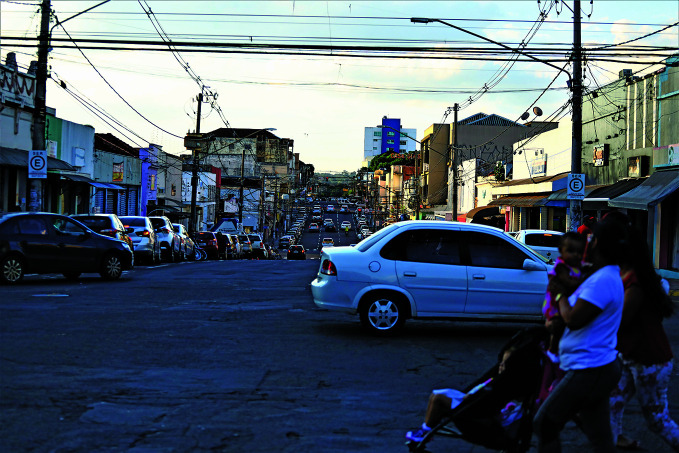
Crossing a busy road in Campo Grande, Brazil before Safe System measures.

Courtesy of PAHO

**Figure:**
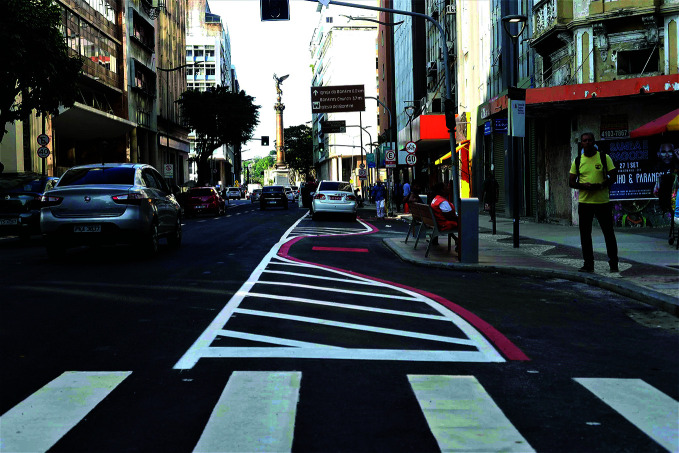
Safe System infrastructure in Salvador, Bahia, Brazil.

PAHO/Jefferson Peixoto

